# Vermicious thermo-responsive Pickering emulsifiers†
†Electronic supplementary information (ESI) available: The theoretical background to the SAXS analysis, gel permeation chromatography analysis of the worms, optical microscopy images and laser diffraction analysis of water droplets. See DOI: 10.1039/c5sc00598a


**DOI:** 10.1039/c5sc00598a

**Published:** 2015-05-07

**Authors:** K. L. Thompson, L. A. Fielding, O. O. Mykhaylyk, J. A. Lane, M. J. Derry, S. P. Armes

**Affiliations:** a Department of Chemistry , University of Sheffield , Brook Hill, Dainton Building , Sheffield , UK S3 7HF . Email: o.mykhaylyk@sheffield.ac.uk ; Email: s.p.armes@sheffield.ac.uk; b Department of Chemical and Biological Engineering , The University of Sheffield , Mappin Street , Sheffield , UK S1 3JD

## Abstract

Thermo-responsive vermicious (or worm-like) diblock copolymer nanoparticles prepared directly in *n*-dodecane *via* polymerisation-induced self-assembly (PISA) were used to stabilise water-in-oil Pickering emulsions. Mean droplet diameters could be tuned from 8 to 117 μm by varying the worm copolymer concentration and the water volume fraction and very high worm adsorption efficiencies (∼100%) could be obtained below a certain critical copolymer concentration (∼0.50%). Heating a worm dispersion up to 150 °C led to a worm-to-sphere transition, which proved to be irreversible if conducted at sufficiently low copolymer concentration. This affords a rare opportunity to directly compare the Pickering emulsifier performance of *chemically identical* worms and spheres. It is found that the former nanoparticles are markedly more efficient, since worm-stabilised water droplets are always smaller than the equivalent sphere-stabilised droplets prepared under identical conditions. Moreover, the latter emulsions are appreciably flocculated, whereas the former emulsions proved to be stable. SAXS studies indicate that the mean thickness of the adsorbed worm layer surrounding the water droplets is comparable to that of the worm cross-section diameter determined for non-adsorbed worms dispersed in the continuous phase. Thus the adsorbed worms form a monolayer shell around the water droplets, rather than ill-defined multilayers. Under certain conditions, demulsification occurs on heating as a result of a partial worm-to-sphere morphological transition.

## Abstract 2

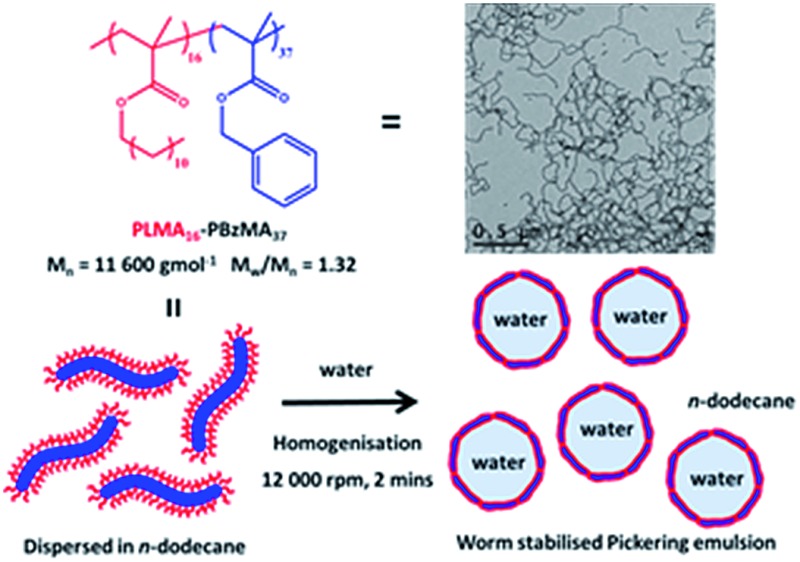
Thermo-responsive vermicious (or worm-like) diblock copolymer nanoparticles prepared directly in *n*-dodecane are used to stabilise water-in-oil Pickering emulsions.

## Introduction

Water or oil droplets stabilised by solid particles are known as Pickering emulsions and have been recognised since the turn of the last century.^1^ They offer various advantages over conventional surfactant-stabilised emulsions, such as enhanced stability towards droplet coalescence, reduced foam problems and low toxicity.^2^,^3^ Many types of particles can act as Pickering emulsifiers, with particle wettability being the most important parameter for determining emulsion type and stability. Spherical particles are utilised in the vast majority of cases,^4^–^6^ and the energy of attachment of an individual particle adsorbed at the oil/water interface increases with particle radius.^3^ As far as we are aware, there is only one literature example of *vermicious (or worm-like) diblock copolymer nanoparticles* being used as Pickering emulsifiers.^7^ This is perhaps surprising, because it has been known for several decades that diblock copolymer ‘worms’ can be formed spontaneously in solution *via* self-assembly.^8^,^9^ Consider a flexible worm of contour length *L* and mean worm width 2*R*, where *L* ≫ 2*R*. Such worms can be prepared from the 1D fusion of spherical precursors of approximate radius *R* at relatively high solids *via* polymerisation-induced self-assembly (PISA).^10^ To a first approximation, a worm formed from the 1D fusion of *n* spheres is expected to have an energy of attachment *n* times larger than that of a single precursor sphere. In addition, cooperativity is expected to play an important role. Thus it is expected to cost significantly more energy to detach a highly anisotropic worm (for which *L*/2*R* > 20) from the oil/water interface compared to the detachment of *n* spheres. Moreover, the specific surface area of such worms is only approximately one-third less that of than the precursor spheres (see ESI†). Hence block copolymer worms offer *significantly enhanced adsorption* compared to spheres, while retaining a *relatively high specific surface area*. Recently the interfacial behavior of anisotropic particles has begun to receive some attention. For example, Noble *et al.*^11^ prepared so-called ‘hairy’ colloidosomes stabilised by relatively large polydisperse polymeric micro-rods^12^ (10–70 μm in length and 0.4–2 μm in width) using a gel-trapping technique. The same microrods have also been used to stabilise aqueous foams.^13^ Vermant and co-workers^14^,^15^ have exploited a mechanical alignment technique to prepare near-monodisperse, micrometer-sized ellipsoidal polystyrene latex particles. When these model ellipsoids were adsorbed at the oil/water interface a strong correlation between the particle aspect ratio and emulsion stability was observed. More specifically, the spherical precursor latex particles proved to be ineffective emulsifiers and a certain minimum aspect ratio was required before effective stabilisation could be achieved. However, the preparation of such ellipsoids does not appear to be readily amenable to scale-up. Cellulose fibres have also been recently explored as Pickering emulsifiers.^16^–^20^ It was found that the fibre aspect ratio dictated the droplet surface coverage.^17^ Relatively short fibres led to a densely-packed layer at the oil/water interface (>80% surface coverage), while longer fibres led to the formation of a more open 2D network (surface coverage ∼40%). Very recently, surface-modified cellulose was utilised in conjunction with non-modified cellulose for the construction of oil-in-water-in-oil double emulsions.^21^ However, surface modification of cellulose fibres involves multi-step protocols and extensive purification, which makes scale-up of such nanoparticles potentially problematic. Thermo-responsive oil-in-water emulsions have also been prepared by grafting poly(*N*-isopropylacrylamide) onto cellulose fibres.^22^ Thermo-responsive foams have also been reported, with fatty acid tube-stabilised foams becoming destabilised upon heating as a result of a tube-to-micelle transition.^23^

**Fig. 1:**
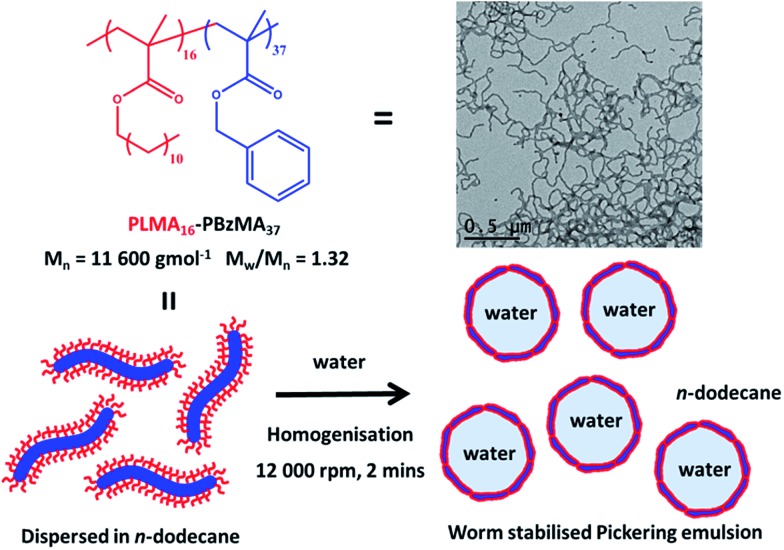
Preparation of Pickering emulsions prepared by homogenisation of PLMA_16_-PBzMA_37_ diblock copolymer worm-like micelles dispersed in *n*-dodecane with an equal volume of water at 12 000 rpm for 2 minutes at 20 °C.

Recent advances in polymerisation-induced self-assembly (PISA) formulations now allow well-defined diblock copolymer worms to be readily prepared on a multi-gram scale at 20% w/w solids.^24^ In particular, linear and cross-linked triblock copolymer worms prepared in aqueous media *via* PISA are generally more effective emulsifiers than spherical nanoparticles.^7^ Such worms are approximately *two orders of magnitude smaller* than the polymeric micro-rods reported earlier.^11^,^12^ Reversible addition–fragmentation chain transfer (RAFT) dispersion polymerisation of benzyl methacrylate (BzMA) in *n*-alkanes using a poly(lauryl methacrylate) macromolecular chain transfer agent (PLMA macro-CTA) enables the reproducible synthesis of PLMA-PBzMA diblock copolymer worms (see Fig. 1).^25^,^26^ Moreover, the worms undergo a worm-to-sphere transition on heating.^26^ This change in morphology is the result of surface plasticisation of the core-forming PBzMA block by the hot solvent, which leads to a subtle reduction in the copolymer packing parameter, *P*.^27^

Herein these highly hydrophobic PLMA_16_-PBzMA_37_ worms are examined as putative Pickering emulsifiers. It is emphasised that their thermo-responsive nature allows conversion into the corresponding spheres, which enables the Pickering performance of worms and spheres to be directly compared using *precisely the same diblock copolymer composition*. Moreover, we examine whether the thermo-sensitivity of the worms can be exploited to obtain *thermo-responsive emulsions*.

## Experimental

### Materials

Monomers were purchased from Sigma-Aldrich (UK) and passed through basic alumina prior to use. *n*-Dodecane (≥90% technical grade), CDCl_3_ and all other reagents were purchased from Sigma-Aldrich (UK) and were used as received, unless otherwise noted. THF and toluene were purchased from Fisher Scientific (UK), while CD_2_Cl_2_ and d_26_-dodecane were purchased from Goss Scientific (UK).

### Synthesis of diblock copolymer worms

PLMA_16_-PBzMA_37_ diblock copolymer worms were prepared as a soft free-standing gel directly in *n*-dodecane *via* polymerisation-induced self-assembly (PISA) at 20% w/w solids according to a previously reported protocol.^26^

### Pickering emulsion preparation

The as-prepared 20% w/w dispersion of PLMA_16_-PBzMA_37_ worms was serially diluted using *n*-dodecane to obtain copolymer concentrations ranging between 0.06 and 2.0 wt%. For the preparation of worm-stabilised Pickering emulsions, a dilute worm dispersion (0.20–3.20 mL) was homogenised directly with deionised water (0.80–3.80 mL) for 2.0 minutes using a IKA Ultra-Turrax T-18 homogeniser with a 10 mm dispersing tool operating at 12 000 rpm. For the corresponding sphere-stabilised emulsions, a 0.50% w/w worm dispersion was first heated to 150 °C in an oil bath for 90 minutes prior to cooling to 20 °C. The resulting dispersion of PLMA_16_-PBzMA_37_ spheres (2.0 mL) was homogenised with water (2.0 mL) as above.

### Characterisation

#### THF GPC

Molecular weight distributions were assessed by gel permeation chromatography (GPC) using THF eluent. The THF GPC system was equipped with two 5 μm (30 cm) mixed C columns, a LC20AD ramped isocratic pump and a WellChrom K-2301 refractive index detector operating at 950 ± 30 nm. The THF mobile phase contained 2.0 v/v% triethylamine and 0.05 w/v% butylhydroxytoluene (BHT) and the flow rate was fixed at 1.0 mL min^–1^. A series of ten near-monodisperse poly(methyl methacrylate) standards (*M*_p_ values ranging from 1280 to 330 000 g mol^–1^) were used for calibration.

#### ^1^H NMR spectroscopy

^1^H NMR spectra were recorded in either CDCl_3_ or CD_2_Cl_2_ using a Bruker Avance 400 spectrometer operating at 400 MHz.

#### Dynamic light scattering (DLS)

Intensity-average hydrodynamic diameters were obtained by DLS using a Malvern Zetasizer NanoZS instrument at a fixed scattering angle of 173°. Dispersions of 0.01% w/w PLMA_16_-PBzMA_37_ particles in *n*-dodecane were analysed using glass cuvettes and the results were averaged over three consecutive runs. It should be noted that DLS reports intensity-average diameters and implicitly assumes a spherical morphology. Thus the DLS dimensions reported herein for the highly anisotropic worm-like particles are actually ‘sphere-equivalent’ diameters that do not provide accurate information regarding either the mean worm length or the mean worm width. Nevertheless, DLS observations of a relatively larger particle size (and also much greater polydispersity) are a useful indication of the presence of worms (as either a pure phase or as one or more mixed phases). In contrast, the corresponding spheres are significantly smaller and have much lower polydispersities.

#### Transmission electron microscopy

Transmission electron microscopy (TEM) studies were conducted using a Philips CM 100 instrument operating at 100 kV and equipped with a Gatan 1k CCD camera. Diluted block copolymer dispersions (0.50% w/w) were placed on carbon-coated copper grids and exposed to either ruthenium tetraoxide vapor for 7 minutes at 20 °C or 0.75% w/w uranyl formate solution (9 μL) was soaked on the sample-loaded grid for 20 seconds. In each case the heavy metal acts as a stain to improve contrast. The ruthenium(viii) oxide was prepared as follows: ruthenium(iv) oxide (0.30 g) was added to water (50 g) to form a black slurry; addition of sodium periodate (2.0 g) with stirring produced a yellow solution of ruthenium(viii) oxide within 1 min at 20 °C.

#### Optical microscopy

Optical microscopy images were recorded using a Motic DMBA300 digital biological microscope equipped with a built-in camera and analysed using Motic Images Plus 2.0 ML software. Number-average (*D*_n_ or *D*[1,0]) and surface-average (*D*[3,2]) droplet sizes were estimated using Image J software; >300 droplets per sample were measured in each case.

#### Laser diffraction

The volume-average droplet (*D*[4,3]) diameter was determined using a Malvern Mastersizer 2000 instrument equipped with a small volume Hydro 2000SM sample dispersion unit (*ca.* 50 mL), a He–Ne laser operating at 633 nm, and a solid-state blue laser operating at 466 nm. The stirring rate was adjusted to 3000 rpm in order to avoid sedimentation of the emulsion during analysis. After each measurement, the cell was rinsed once with *n*-dodecane. The glass walls of the cell were carefully wiped to avoid cross-contamination and the laser was aligned centrally to the detector prior to data acquisition.

#### Small angle X-ray scattering (SAXS)

SAXS patterns were collected using both a laboratory SAXS instrument (Bruker AXS Nanostar modified with a Xenocs GeniX 3D ultralow divergence X-ray source and a collimator comprising two scatterless slits) and a synchrotron source (station ID02, ESRF, Grenoble, France) equipped with a HiStar area detector and a Rayonix MX170 detector, respectively. SAXS patterns were recorded over a scattering vector (*q*) range of 0.08 nm^–1^ < *q* < 1.6 nm^–1^ and 0.005 nm^–1^ < *q* < 0.12 nm^–1^, respectively using monochromatic X-ray radiation (wavelength *λ* = 0.154 nm and *λ* = 0.1 nm, respectively), where the length of the scattering vector, *q*, is given by *q* = (4π sin *θ*)/*λ* and *θ* is half of the scattering angle. A liquid cell comprising two mica windows (each of 25 μm thickness) separated by a polytetrafluoroethylene spacer of 1.0 mm thickness or a glass capillary (1.7 mm diameter) were used as sample holders for either in-house measurements or synchrotron measurements, respectively. All experiments were performed at 21 °C. Scattering data were reduced using Nika SAS data reduction macros for Igor Pro (integration, normalisation and background subtraction) and further analysed using Irena SAS macros for Igor Pro.^28^ Water and a glassy carbon standard were used for absolute intensity calibration.^29^ SAXS measurements were conducted on 1.0% w/w PLMA_16_-PBzMA_37_ solution in *n*-dodecane and a concentrated water-in-*n*-dodecane emulsion prepared by emulsification of 50 vol% water with 50 vol% of a 1.0% w/w PLMA_16_-PBzMA_37_ worm dispersion in *n*-dodecane at 12 000 rpm for two minutes at 21 °C. Excess non-adsorbed worms were removed from the continuous phase by successive replacement of the supernatants (obtained after allowing the emulsion to stand at room temperature for at least 16 h) with fresh *n*-dodecane. This supernatant replacement protocol was repeated five times. The theoretical background for the structural model used for SAXS data analysis is given in the ESI.†

## Results and discussion

### General remarks

**Fig. 2:**
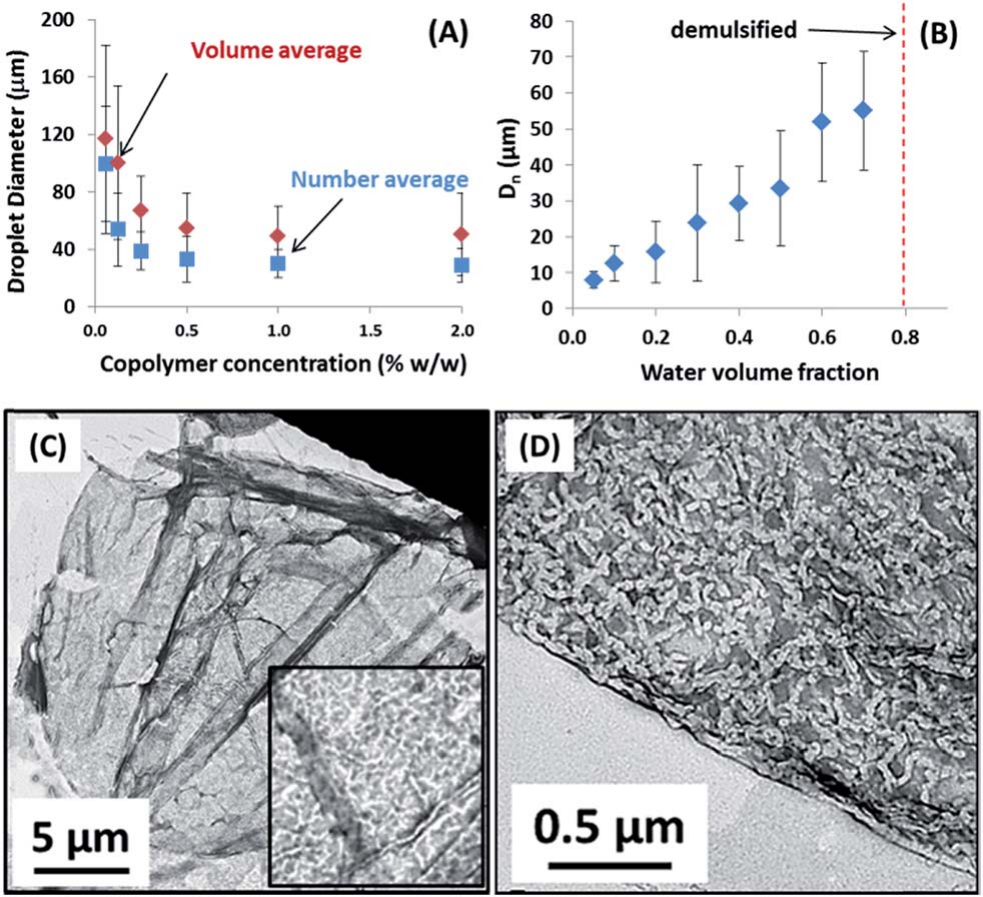
(A) Water droplet diameter as a function of PLMA_16_-PBzMA_37_ worm concentration in *n*-dodecane as determined by optical microscopy (number-average) and laser diffraction (volume-average). (B) Number-average water droplet diameter *vs.* water volume fraction for a fixed copolymer concentration of 0.50% w/w. (C and D) TEM images of the surface of a dried, collapsed Pickering emulsion droplet (using a 0.50% w/w worm concentration and a water volume fraction of 0.50) after evaporation of both the aqueous droplet phase and the *n*-dodecane continuous phase. The PLMA_16_-PBzMA_37_ worms are clearly visible intact at the surface of this dried droplet.

The as-prepared PLMA_16_-PBzMA_37_ worms obtained at 20% w/w solids in *n*-dodecane were diluted using the same solvent. TEM studies confirmed that the worms were both flexible and highly anisotropic, with a mean aspect ratio (*L*/2*R*) greater than 20 being estimated from software analysis of 100 particles (see Fig. 1). In initial high-shear homogenisation studies, the volume fraction of the oil and water phases were fixed at 0.50 and the worm copolymer concentration was systematically varied from 2.0 to 0.06% w/w *via* serial dilution (see Fig. 2A). Stable water-in-oil Pickering emulsions were obtained at all copolymer concentrations investigated, with representative optical micrographs of the aqueous emulsion droplets being shown in the ESI (see Fig. S2†). Gravitational sedimentation of the denser aqueous droplet phase occurred on standing over a time scale of minutes to hours (depending on the droplet diameter). However, excellent stability towards droplet coalescence was observed, since essentially no change in mean droplet diameter with time was detected after standing at 20 °C for several months.

### Particle size analysis of emulsion droplets

Both optical microscopy and laser diffraction studies indicate an inverse relationship between the mean droplet diameter and the worm copolymer concentration (see Fig. 2a; the former technique reports a number-average diameter and hence always undersizes relative to the latter). Similar trends have been observed for various spherical Pickering emulsifiers.^30^ Recent work with diblock copolymer vesicles^31^ has highlighted their rather delicate nature when prepared using an aqueous PISA formulation. In this case, high shear homogenisation led to *in situ* vesicle dissociation, with the resulting individual copolymer chains then acting as a polymeric surfactant for the oil droplets. Such vesicle degradation was readily apparent, since the mean droplet diameter was essentially independent of the copolymer concentration. This is because only a very low copolymer concentration is required to stabilise the relatively large emulsion droplets if the copolymer is present as soluble chains. Chemical cross-linking of the copolymer vesicles was required to prevent vesicle dissociation during homogenisation. Such covalent stabilisation led to genuine Pickering emulsions that exhibited the expected concentration-dependent variation in mean droplet diameter.

### Structure of adsorbed worms and adsorption efficiency

For the linear PLMA_16_-PBzMA_37_ worms described herein, there is no evidence for *in situ* worm dissociation during homogenisation. The TEM images of dried droplets shown in Fig. 2C provide strong evidence that the PLMA_16_-PBzMA_37_ worms remain intact during high shear homogenisation and subsequently form a dense layer on the surface of the aqueous droplets. The adsorption efficiency of the worms on the water droplets was estimated using a previously reported turbidimetric assay (see ESI† for further details).^31^ At copolymer concentrations below 0.50% w/w, essentially all the worms adsorb at the oil/water interface (see Table S1†). This critical copolymer concentration also corresponds to the limiting OM droplet diameter of 30 μm observed under these conditions. At higher copolymer concentrations (1–2% w/w), lower worm adsorption efficiencies are observed. An increase in mean droplet diameter was observed at a fixed worm copolymer concentration of 0.50% w/w when varying the water volume fraction from 0.05 to 0.70. Phase inversion is often observed in the literature when the volume fraction of the preferred droplet phase (in this case, water) exceeds that of the continuous phase.^3^ In contrast, a high internal phase Pickering emulsion is obtained at water volume fractions of 0.60–0.70 in the present work, see Fig. 2B. However, above a critical volume fraction of 0.70, stable water droplets cannot be obtained; instead, complete demulsification is observed. This suggests that the PLMA_16_-PBzMA_37_ worms have a strong preference for the oil phase, which is not surprising given their highly hydrophobic character. The worm adsorption efficiency is reduced when the water volume fraction falls below 0.50, since there are now more worm-like particles available for adsorption.

### Pickering emulsifier performance of worms *vs.* spheres

**Fig. 3:**
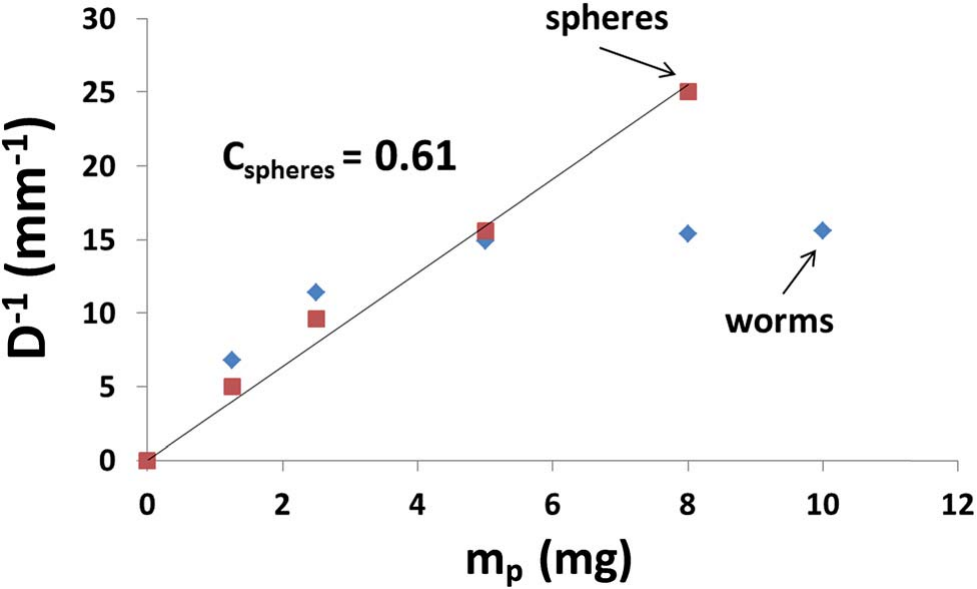
Effect of varying the copolymer particle mass *m*_p_ on the droplet diameter for two series of water-in-*n*-dodecane emulsions stabilised using (a) PLMA_16_-PBzMA_37_ spheres (red squares) and (b) PLMA_16_-PBzMA_37_ worms (blue diamonds). Note the deviation from linearity for the latter particles.

Recently, we reported that PLMA_16_-PBzMA_37_ worms prepared in *n*-dodecane display thermo-responsive behavior.^26^ On heating, worms are converted into spheres as a result of surface plasticisation of the PBzMA block by the hot solvent. At sufficiently high copolymer concentration (>5% w/w), this order–order transition is essentially reversible, as judged by TEM, SAXS and rheology studies.^26^ However, this worm-to-sphere transformation is *irreversible* when heating dilute worm dispersions (∼1.0% w/w). The spheres represent a kinetically-trapped morphology.^32^ This is because the self-assembly of an individual worm from multiple spheres is a highly cooperative process that is disfavored at low copolymer concentrations.^26^ Thus the copolymer morphology remains spherical over experimentally accessible time scales after cooling to 20 °C. This thermal behavior provides a rare opportunity to directly compare the Pickering emulsifier performance of highly anisotropic worms with chemically-identical spheres with essentially the same wettability. Two separate sets of experiments were conducted to assess whether the worm-stabilised Pickering emulsions also exhibited thermo-responsive behavior. First, dilute copolymer worm dispersions (0.06–1.00% w/w) were heated to 150 °C for 90 min and then cooled to 20 °C prior to homogenisation with an equal volume of water. In this case, the worms were expected to become ‘trapped’ in their spherical morphology prior to adsorption at the o/w interface. Indeed, this thermal treatment led to a significant reduction in the sphere-equivalent DLS particle diameter and polydispersity (PDI) from 150 nm (PDI = 0.28) to 30 nm (PDI = 0.03). TEM studies confirmed the expected worm-to-sphere transition (see Fig. S5†). It is emphasised that *the resulting PLMA*_*16*_*-PBzMA*_*37*_*spherical nanoparticles have precisely the same copolymer composition as the original worms*, which enables the effect of copolymer morphology on Pickering emulsifier performance to be assessed. Deploying the spherical nanoparticles at copolymer concentrations of 0.06–1.0% w/w invariably led to the formation of w/o emulsions. This is an interesting observation given that Vermant and co-workers reported that spherical polystyrene latex particles do not stabilise emulsions as effectively as ellipsoidal latexes with the same surface chemistry.^14^ However, laser diffraction studies of the emulsions prepared using the PLMA_16_-PBzMA_37_ spheres indicated important differences compared to worm-stabilised emulsions. The former emulsions proved to be flocculated since volume-average diameters determined by laser diffraction were significantly larger than the number-average diameters estimated from optical microscopy, see ESI Fig. S6.† In contrast, the worm-stabilised emulsion droplets showed no signs of flocculation. In addition, a significantly smaller mean droplet diameter (*D*) was always observed for the worms when working above a certain critical copolymer mass (*m*_p_), see Fig. 3.

As shown in Fig. 2A, the mean droplet diameter can be controlled by the copolymer concentration (and hence copolymer mass). Assuming that all the particles are adsorbed at the oil/water interface, which is the case for copolymer concentrations ≤0.50% w/w, *D* is calculated for spheres using eqn (1) and for worms using eqn (2):^16^,^33^
1
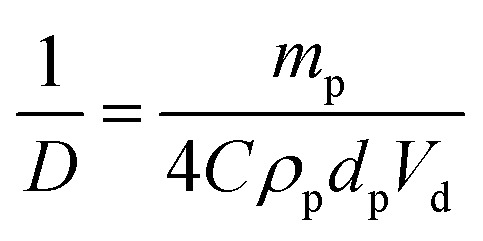

2
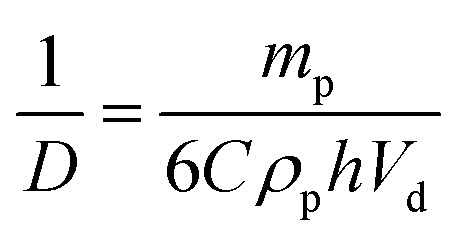
Here *m*_p_ is the particle mass, *ρ*_p_ is the particle density (taken to be that of the PBzMA core-forming block, or 1.15 g cm^–3^), *d*_p_ is the sphere diameter, *h* is the worm thickness, *V*_d_ is the volume of the dispersed (droplet) phase and *C* is the fractional surface coverage (*i.e.* the fraction of the droplet interfacial area actually covered by adsorbed particles). By plotting the inverse of the mean droplet diameter *D* as a function of *m*_p_, *C* can be deduced from the gradient, see Fig. 3. [N.B. the flocculated nature of the sphere-stabilised emulsions precluded using laser diffraction, so the mean droplet diameters used for this plot were estimated by optical microscopy.] A linear relationship is usually observed for spherical particles,^33^ indicating that *C* is constant over the entire droplet size range. As expected, sphere-stabilised droplets exhibited this relationship, with a corresponding fractional surface coverage of 0.61. This is physically reasonable for close-packed spheres on a curved 2D interface.^34^ However, when the same plot is constructed for the worm-stabilised emulsions, a marked deviation from linearity is observed at high values of *m*_p_. This suggests an increase in droplet surface coverage at higher copolymer concentration. Such behavior has been recently reported for Pickering emulsifiers based on anisotropic bacterial cellulose nanofibres.^16^ This increase in fractional surface coverage can be directly related to the anisotropic nature of the worms. At low worm concentrations, their high aspect ratio leads to rather loosely-packed particles at submonolayer coverage, while a much more densely packed worm layer is formed at relatively high worm concentrations. It is emphasised that the linear worms used in the present study are much more flexible than the rigid rod-like bacterial cellulose particles used by Kalashnikova *et al.*^16^ This suggests that particle anisotropy, rather than ‘stiffness’, is responsible for the similar interfacial behavior observed in both cases.

### SAXS studies of worm-stabilised emulsions

**Fig. 4:**
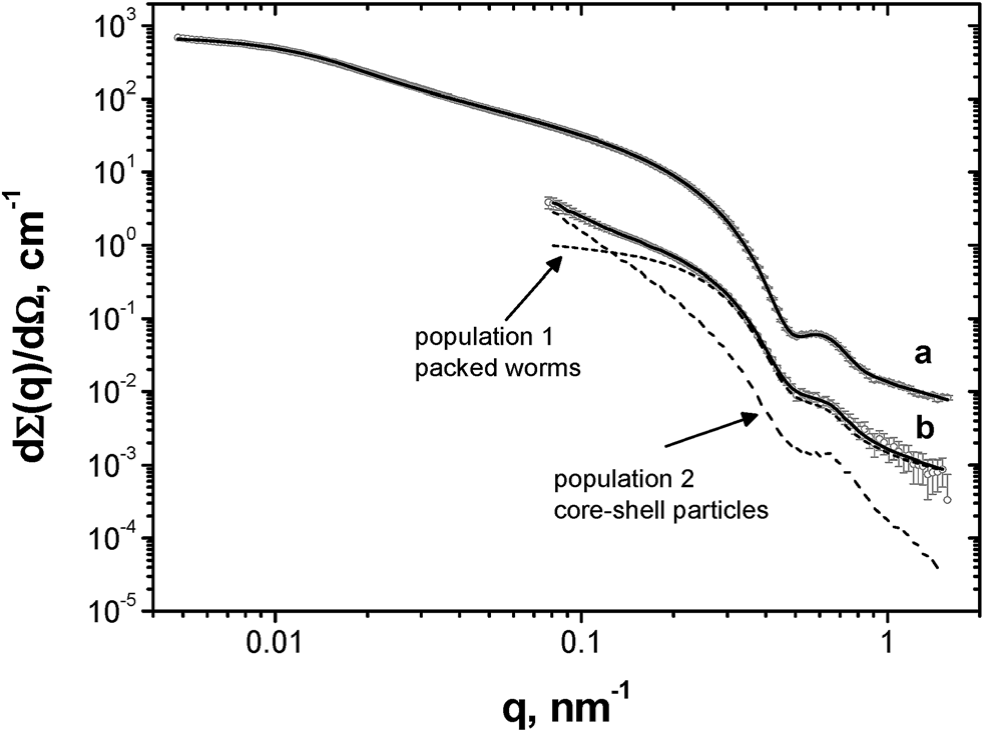
Experimental SAXS data (circles) and fitting curves (solid lines) of (a) a dilute (1.0% w/w) PLMA_16_-PBzMA_37_ worm dispersion in *n*-dodecane and (b) an emulsion comprising aqueous droplets in *n*-dodecane stabilised by PLMA_16_-PBzMA_37_ worms. Two dashed curves indicate the relative contributions of each population to model 2 (adsorbed worms and core–shell particles) to the total scattering of the curve fit.

Unlike TEM, SAXS studies (see Fig. 4) enable the structure of the adsorbed worm layer surrounding the water droplets to be assessed *in situ*. In view of our TEM observations (Fig. 2), laser diffraction droplet diameter and previously reported SAXS data obtained for a PLMA_16_-PBzMA_37_ worm dispersion in *n*-dodecane,^26^ it was assumed that the SAXS patterns corresponded to (i) a pure worm phase (Fig. 4a) for the dilute PLMA_16_-PBzMA_37_ dispersion and (ii) spherical aqueous droplets stabilised by a layer of adsorbed worms for the concentrated water-in-*n*-dodecane emulsion (Fig. 4b). Thus data analysis utilised two models (see ESI†): a worm-like micelle model^35^,^36^ (model 1) and a two population core–shell model comprising a particulate shell formed by the adsorbed worms (model 2).

In contrast to the original worm dispersion (Fig. 4a), the scattering pattern for the emulsion (Fig. 4b) exhibits an upturn in scattering intensity at low *q* (below 0.15 nm^–1^), indicating the presence of large objects, *i.e.* the aqueous droplets that are observed by optical microscopy and laser diffraction (Fig. 1). The X-ray data recorded at high *q* (0.5–1.6 nm^–1^) are somewhat noisy because the worm volume fraction is relatively low (only ∼0.00056, see Table 1).^37^

**Table 1:** Structural parameters obtained by SAXS analysis of a 1.0% w/w PLMA_16_-PBzMA_37_ worm dispersion in *n*-dodecane (model 1) and a water-in-*n*-dodecane emulsion (water volume fraction = 0.50) stabilised using the same PLMA_16_-PBzMA_37_ worms (model 2)^*a*^

Parameters	Model 1	Model 2
**Population 1**		
Worm contour length, *L*_w_, nm	591 ± 9	591^*a*^
Kuhn length, *b*_w_, nm	194 ± 6	194^*a*^
Worm core cross-section radius, *R*_sw_, nm	5.9 ± 0.01	5.9 ± 0.1
*R*_sw_ standard deviation, *σ*_11_, nm	0.74 ± 0.01	1.0 ± 0.08
Solvent volume fraction in the worm cores, *x*_sol_	∼0	∼0
Radius of gyration of the corona block, *R*_g_, nm	1.3 ± 0.1	1.1 ± 0.1
Copolymer volume fraction, *c*_1_	0.0069 ± 0.00004	0.00056 ± 0.00004
Second virial coefficient (packing parameter), *A*_2_ × 10^16^	—	1.68 ± 0.42

**Population 2**		
Core–shell radius, *R*_cs_, nm	—	24 500^*a*^
*R*_cs_ standard deviation, *σ*_21_, nm	—	10 500^*a*^
Shell thickness, *T*_cs_, nm	—	12 ± 1.7
*T*_cs_ standard deviation, *σ*_22_, nm	—	2.0^*b*^
Core–shell particles volume concentration, *c*_2_	—	0.251 ± 0.005

^*a*^The indicated parameters were determined independently and were fixed during data fitting.

^*b*^The standard deviation for *T*_cs_ is directly related to that of *R*_sw_ (*σ*_22_ = 2*σ*_11_).

No structure factor was required for satisfactory data analysis of the 1.0% w/w worm dispersion in *n*-dodecane (see Fig. 4a, eqn (S3)† and Table 1 for further details). In contrast, a structure factor for the worm population had to be incorporated (see eqn (S5) and (S6)† and Table 1 for further details) in order to obtain a satisfactory data fit when using the two-population model. This suggests that the worms become interacting, which is consistent with their adsorption onto the surface of the aqueous droplets in the form of a network (Fig. 2c and d).

In addition, the modest increase in the standard deviation of the worm cross-section radius (*σ*_11_) and reduction in the radius of gyration of the corona block (*R*_g_) observed for the emulsion (model 2, Table 1) relative to the original worms (model 1, Table 1) may indicate some perturbation in the circular cross-section of the adsorbed worms at the oil/water interface. The SAXS fitting parameters confirm that the shell thickness of the core–shell particles, *T*_cs_, is comparable to the cross-section worm diameter, 2*R*_sw_ (where *R*_sw_ is the worm cross-section radius), see Table 1. To a good first approximation, this suggests that the worms adsorb onto the water droplets to form a *monolayer* shell, rather than a relatively thick multilayer shell. However, it is emphasised that the scattering intensities originating from both populations in model 2 have overlapping minima at *q* ∼ 0.50 nm^–1^ associated with either the shell thickness or with the worm cross-section (Fig. 4b). If a worm monolayer is formed around the emulsion droplets, then the shell dimensions are comparable to the worm cross-section, so these two associated scattering features occur at approximately the same *q* value (and so cannot be resolved). Thus it follows that application of more sophisticated contrast variation techniques (for example, using small-angle neutron scattering, or SANS) in order to attempt to delineate the scattering contribution of the worms from that of the worm-stabilised aqueous droplets are unlikely to be more informative. The ratio of the volume fraction of the worm-stabilised aqueous droplets to that of the adsorbed worms present in the emulsion can be expressed as a ratio of the core–shell particle volume to the shell volume. Hence this volume ratio, *V*, can be estimated as follows from the fitted SAXS parameters (model 2, Table 1):
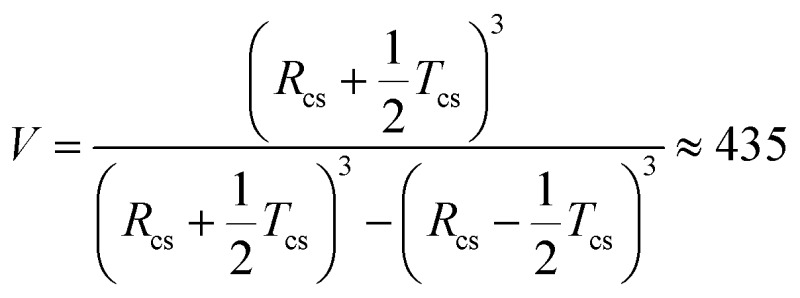


This estimate is roughly comparable to the ratio of the fitted volume fractions of the core–shell particles and the worm micelles, *c*_2_/*c*_1_ ≈ 261 (see Table 1). Some discrepancy between these values is to be expected given the relatively large errors in *c*_1_ and *c*_2_ (see Table 1) and also because the aqueous droplet diameter was determined by laser diffraction, rather than measured directly using SAXS.

Curve-fitting the SAXS data also suggests that the volume fraction of the aqueous droplets (core–shell particles), *c*_2_, is 0.25 (see Table 1). Given the various experimental uncertainties, this value is in fair agreement with the water volume fraction of 0.50 used for emulsion preparation. The discrepancy is most likely to arise from uncertainty regarding the true nature of the droplet size distribution (a Gaussian distribution was assumed in the SAXS analysis), the relatively broad range in droplet size indicated by the laser diffraction data (see last column in Table S1†) and the fact that the volume-average diameter reported by the latter technique was used in the SAXS analysis (rather than using SAXS to directly determine the core–shell particle size distribution).

Notwithstanding the above approximations, the SAXS data presented in Fig. 4 provide good evidence for the formation of an *adsorbed worm monolayer* on the surface of the water droplets, which is consistent with the 2D worm network structure observed by TEM (see Fig. 2).

### Thermo-responsive emulsions

A second set of experiments were conducted whereby the sphere- and worm-stabilised emulsions were each heated in turn up to 95 °C for 90 min in order to observe any change in emulsion stability [N.B. 95 °C was selected to avoid significant evaporation of the aqueous droplet phase]. Demulsification occurred to varying extents, depending on both the copolymer concentration and also whether the droplets were stabilised by worms or spheres (see Table S2†). Worm-stabilised droplets generally proved to be more stable after heating. For example, at a copolymer concentration of 0.13% w/w, only 17% demulsification was observed for worms, compared to complete demulsification for sphere-stabilised droplets. Both types of emulsions underwent complete phase separation at a copolymer concentration of 0.06% w/w (*e.g.* see Fig. S7†). The original emulsions could be reformed *via* rehomogenisation at 20 °C. However, the heat-treated worms (DLS diameter = 45 nm; PDI = 0.17) were significantly shorter than the original worms (DLS diameter = 150 nm; PDI = 0.28). The volume-average droplet diameter increased after heating the worm-stabilised emulsions, particularly when using higher copolymer concentrations. Long worms are more strongly adsorbed than short worms and also provide a more effective barrier to droplet coalescence, especially when present at low surface coverage. Hence the reduction in mean worm length that occurs on heating leads to limited droplet coalescence. At lower copolymer concentrations, complete demulsification is observed for the worm-stabilised emulsions at 95 °C. Again, this is related to the reduction in mean worm length. However, the fact that the sphere-stabilised water droplets also coalesce on heating was not anticipated. This observation suggests that the known partial plasticisation^13^ of the core-forming PBzMA block at 95 °C causes appreciable swelling of the spheres, which lowers the particle contact angle and hence induces interfacial desorption. A similar mechanism has been invoked to account for the demulsification that occurs on addition of acid when using pH-responsive polymer particles as Pickering emulsifiers.^38^–^40^

## Conclusions

In summary, water-in-oil Pickering emulsions can be stabilised using highly anisotropic PLMA_16_-PBzMA_37_ diblock copolymer worms prepared *via* PISA directly in the oil phase. TEM studies indicate that these linear worms are stable to the high shear emulsification protocol.^16^ The number-average aqueous droplet diameter can be tuned from 10 to 100 μm diameter by varying the emulsification conditions. Turbidimetry studies indicate that essentially all of the worms are adsorbed onto the water droplets at a copolymer concentration of 0.50% w/w or below. Heating dilute worm dispersions in *n*-dodecane up to 150 °C leads to a worm-to-sphere transition, which proved to be irreversible if conducted at sufficiently low copolymer concentration. Thus this affords a rare opportunity to directly compare the Pickering emulsifier performance of *chemically identical* worms and spheres. However, analysis of the emulsion droplet size and stability with respect to the mass of adsorbed copolymer indicates *qualitatively different behavior for the spheres and worms*. The anisotropic nanoparticles proved to be markedly more efficient, since worm-stabilised water droplets are always smaller than the equivalent sphere-stabilised droplets prepared under identical conditions. Moreover, the former emulsions are appreciably flocculated, whereas the latter emulsions proved to be stable. SAXS studies indicate that the thickness of the adsorbed worm layer on the water droplets is comparable to that of the worm cross-section diameter determined for non-adsorbed worms dispersed in the continuous phase. Thus this provides direct experimental evidence for the adsorbed worms forming a 2D network around the water droplets, with a mean thickness that corresponds to approximately monolayer coverage, rather than ill-defined multilayers. Both worm- and sphere-stabilised emulsions undergo spontaneous demulsification on heating up to 95 °C. This thermo-responsive behaviour is related to the plasticisation of the PBzMA core-forming block by the hot *n*-dodecane.^26^ This induces a partial worm-to-sphere transition and hence causes copolymer desorption from the oil/water interface, leading to rapid droplet coalescence. Thus the thermo-responsive nature of these copolymer worms is conferred on the worm-stabilised water-in-oil Pickering emulsion. When combined with our recent complementary study,^7^ the present work confirms that block copolymer worms can offer a *generic* strategy for the preparation of Pickering emulsions. This approach can be exploited to prepare Pickering *double emulsions*, as recently reported elsewhere.^41^

## Supplementary Material

Supplementary information
