# Supplementary material for: Vermicious thermo-responsive Pickering emulsifiers
Source: Chem Sci. 2015 May 7;6(7):4207–14. doi: 10.1039/c5sc00598a (PMC5707463; doi:10.1039/c5sc00598a)
Supplement: Supplementary file 1 [file SC-006-C5SC00598A-s001.pdf]

Electronic Supplementary Material (ESI) for Chemical Science.
This journal is © The Royal Society of Chemistry 2015

Supporting Information for

Vermicious Thermo-responsive Pickering Emulsifiers

*Kate L. Thompson, Lee A. Fielding, Oleksandr O. Mykhaylyk, Jacob A. Lane, Matthew J. Derry, and Steven P. Armes**

Theoretical background for SAXS analysis

In view of our TEM observations (Figure 2) and previously reported SAXS data obtained for a $PLMA_{16}$-$PBzMA_{37}$ solution in *n*-dodecane[1], it was assumed that the SAXS patterns corresponded to (i) a pure worm phase (Figure 4a) for the dilute $PLMA_{16}$-$PBzMA_{37}$ dispersion and (ii) spherical aqueous droplets stabilised by a layer of adsorbed worms for the concentrated water-in-*n*-dodecane emulsion (Figure 4b). Thus data analysis utilized two models: a worm-like micelle model[2, 3] (model 1) and a core-shell model comprising a particulate shell formed by the adsorbed worms (model 2).

In general, the X-ray intensity scattered by a system composed of *n* different (non-interacting) populations of polydisperse objects [usually described by the differential scattering cross-section per unit sample volume, $d\Sigma(q)/d\Omega$] can be expressed as

$$\frac{d\Sigma(q)}{d\Omega} = \sum_{l=1}^{n} S_l(q) P_l \int_0^{\infty} \dots \int_0^{\infty} F_l(q, r_{l1}, \dots, r_{lk})^2 \Psi_l(r_{l1}, \dots, r_{lk}) dr_{l1} \dots dr_{lk} \qquad \text{S1}$$

where $F_l(q, r_{l1}, \dots, r_{lk})$ is the form factor, $\Psi_l(r_{l1}, \dots, r_{lk})$ is the distribution function, P_l is the number density per unit volume and $S_l(q)$ is the structure factor of the lth population in the system. $r_{l1}, \dots, r_{lk}$ is a set of *k* parameters describing the structural morphology of the lth population.

In terms of Eq. S1, a dispersion of block copolymer worms can be described as a single population system ($n = 1$). The form factor for such anisotropic nano-objects can be expressed in terms of semi-flexible chains with a circular cross-section[2]

$$F_1(q, R_{sw}) = N_w^2 \beta_s^2 F_{sw}(q) + N_w \beta_c^2 F_c(q, R_g) + N_w (N_w - 1) \beta_c^2 S_{cc}(q) + 2 N_w^2 \beta_s \beta_c S_{sc}(q) \qquad \text{S2}$$

where the X-ray scattering length contrasts for the core and corona blocks are given by $\beta_s = V_s(\xi_s - \xi_{sol})$ and $\beta_c = V_c(\xi_c - \xi_{sol})$, respectively. Here ξ_s, ξ_c, and ξ_{sol} are the X-ray scattering length

densities of the core block [ξ_{PBzMA} = 10.38 × 10^{10} cm^{-2}], the corona block (ξ_{PLMA} = 11.37 × 10^{10} cm^{-2}) and the solvent ($\xi_{n\text{-}dodecane}$ = 7.63 × 10^{10} cm^{-2}), respectively. V_s and V_c are the volumes of the core block ($V_{PBzMA37}$ = 9.4 nm^3) and the corona block (V_{PLMA16} = 5.6 nm^3), respectively. The volumes were obtained from $V = \frac{M_w}{N_A \rho}$ using solid-state homopolymer densities determined by helium pycnometry (ρ_{PBzMA} = 1.15 g cm^{-3} and ρ_{PLMA} = 1.20 g cm^{-3}). The self-correlation term for the worm micelle core with radius R_{sw}, $F_{sw}(q) = F_{worm}(q, L_w, b_w) A^2_{cs\_worm}(q, R_{sw})$, is a product of a core cross-section term $F_{cs\_worm}(q, R_{sw}) = A^2_{cs\_worm}(q, R_{sw}) = \left[2\frac{J_1(qR_{sw})}{qR_{sw}} \right]^2$, where J_1 is the first-order Bessel function of the first kind, and the form factor for self-avoiding semi-flexible chains representing the worm is given by $F_{worm}(q, L_w, b_w)$, where b_w is the worm Kuhn length and L_w is the mean worm contour length. A complete expression for the chain form factor can be found elsewhere[3] (Eq. 26 of this reference with the formalism described therein was used in the present work). The self-correlation term of the corona block in Eq. (S2) is given by the Debye function $F_c(q, R_g) = \frac{2[\exp(-q^2R_g^2) - 1 + q^2R_g^2]}{q^4R_g^4}$. The interference cross-term between the worm-like micelle core and the coronal stabiliser chains is expressed as: $S_{sc}(q) = \psi(qR_g) A_{cs\_worm} J_0[q(R_{sw} + R_g)] F_{worm}(q, L_w, b_w)$, where $\psi(qR_g) = \frac{1 - \exp(-q^2R_g^2)}{q^2R_g^2}$ is the form factor amplitude of the corona chain, R_g is the radius of gyration of the corona block (PLMA), and J_0 is the zero-order Bessel function of the first kind. The interference term between the worm corona chains is expressed as: $S_{cc}(q) = \psi^2(qR_g) J_0^2[q(R_{sw} + R_g)] F_{worm}(q, L_w, b_w)$. The mean aggregation number of the worms is $N_w = (1 - x_{sol}) \frac{\pi R_{sw}^2 L}{V_s}$, where x_{sol} is the solvent volume fraction within the worm cores.

A Gaussian distribution for the worm core radius (with a mean radius R_{sw} and a standard deviation σ_{11}) is assumed for model 1 such that: $\Psi_1(r_{11}) = \frac{1}{\sqrt{2\pi\sigma_{11}^2}} e^{-\frac{(r_{11} - R_{sw})^2}{2\sigma_{11}^2}}$. Thus, the number density in Eq. S1 is

expressed as $P_1 = \frac{c_1}{\int_0^\infty V(r_{11})\Psi_1(r_{11})dr_{11}}$, where c_1 is the volume fraction of copolymer chains forming the worms. A dilute worm dispersion has been assumed in the SAXS analysis, so the structure factor for model 1 is set to unity [$S_1(q) = 1$]. Thus, Eq. S1 for model 1 can be rewritten as

$$\frac{d\Sigma(q)}{d\Omega} = P_1 \int_0^\infty F_1(q, r_{11})^2 \Psi_1(r_{11}) dr_{11} \quad \text{S3}$$

where R_{sw} used in Eq. S2 for the expression of the form factor is replaced by r_{11} to account for the size distribution of the worm core radius.

In order to construct a structural model for the SAXS analysis of aqueous emulsion droplets (stabilised by $PLMA_{16}$-$PBzMA_{37}$ worms) in *n*-dodecane, a previously used formalism for core-particulate shell spherical particles has been employed in this work. The emulsion droplets composed of aqueous cores and a particulate shell comprising adsorbed worms is reminiscent of the core-particulate shell particles previously reported[4], where it was demonstrated that SAXS patterns can be successfully fitted using a two-population model represented by the superposition of two scattering patterns, corresponding to core-shell spherical particles and particles forming a particulate shell respectively.

Considering these previous results[4], it was assumed that the SAXS patterns can be represented as a sum of scattering signals generated by two populations (n = 2 in Eq. S1): worms forming the particulate shell (the first population, l = 1 in Eq. S1) and core-shell particles (the second population, l = 2 in Eq. S1). The terms for the two-population model used in this work can be expressed as follows. The form factor for the first population is identical to that used for model 1 (Eq. S2). The form factor for the second population, corresponding to the core-shell particles, is given by[5]:

$$F_2(q, R_{cs}, T_{cs}) = V_{total}(\xi_{shell} - \xi_{sol}) f[q(R_{cs} + \frac{1}{2}T_{cs})] + V_{core}(\xi_{core} - \xi_{shell}) f[q(R_{cs} - \frac{1}{2}T_{cs})] \quad \text{S4}$$

where R_{cs} is the distance from the centre of the particle to the middle of the shell and T_{cs} is the shell thickness. $V_{total} = \frac{4}{3}\pi(R_{cs} + \frac{1}{2}T_{cs})^3$ and $V_{core} = \frac{4}{3}\pi(R_{cs} - \frac{1}{2}T_{cs})^3$ are volumetric parameters for the core-shell

particles. ξ_{core}, ξ_{shell} and ξ_{sol} are the scattering length densities for the aqueous droplets [ξ_{H2O} = 9.42 × 10^{10} cm^{-2}], the particulate worm shell [volume-averaged scattering length density of the worms $\bar{\xi}_{worm} = (\xi_{PLMA}V_{PLMA16} + \xi_{PBzMA}V_{PBzMA37})/(V_{PLMA16} + V_{PBzMA37})$ = 10.75 × 10^{10} cm^{-2}] and the solvent ($\xi_{n\text{-}dodecane}$ = 7.63 × 10^{10} cm^{-2}), respectively. Function $f(x)$ is a normalized form factor for a homogeneous sphere:

$$f(x) = \frac{3[\sin(x) - x\cos(x)]}{(x)^3}.$$

As for model 1, a Gaussian distribution for the worm core radius (with a mean radius R_{sw} and an associated standard deviation σ_{11}) is used for the first population of model 2. For the second population, a Gaussian distribution is also assumed for both the core-shell particle radius (with a mean radius R_{cs} and a standard deviation σ_{21}) and the shell thickness (with a mean thickness T_{cs} and a standard deviation σ_{22}): $\Psi_2(r_{21}, r_{22}) = \frac{1}{\sqrt{2\pi\sigma_{21}^2}} e^{-\frac{(r_{21}-R_{cs})^2}{2\sigma_{21}^2}} \frac{1}{\sqrt{2\pi\sigma_{22}^2}} e^{-\frac{(r_{22}-T_{cs})^2}{2\sigma_{22}^2}}$. Thus, the number density of the second population in Eq. S1 is expressed as $P_2 = \frac{c_2}{\int_0^\infty V(r_{21}, r_{22})\Psi_2(r_{21}, r_{22})dr_{21}dr_{22}}$, where c_2 is the volume fraction of core-shell particles (i.e., worm-stabilised aqueous droplets) in the emulsion. Although the droplet concentration is high, no structure factor is required for the second population of model 2 over the q range of interest, hence $S_1(q) = 1$. Thus, Eq. S1 for model 2 can be rewritten as:

$$\frac{d\Sigma(q)}{d\Omega} = S_1(q)P_1\int_0^\infty F_1(q, r_{11})^2\Psi_1(r_{11})dr_{11} + P_2\int_0^\infty F_2(q, r_{21}, r_{22})^2\Psi_2(r_{21}, r_{22})dr_{21}dr_{22} \qquad \text{S5}$$

where R_{cs} and T_{cs} used in Eq. S4 for the expression of the core-shell form factor are substituted by r_{21} and r_{22} in order to account for the size distributions of both the core-shell radius and the shell thickness. Since the worms forming the particulate shell are expected to be quite densely packed (see Figure 2c), a structure factor for the first population, $S_1(q)$, is incorporated into the model. In principle, a structure factor based on the polymer reference interaction site model (PRISM) proposed for interacting worms could be used in this case.[6] However, there was no opportunity in the present SAXS study to obtain the

appropriate PRISM parameters to describe the effect of copolymer concentration on the scattering profile. Instead, a simplified approach based on a virial expansion[7] was used to account for the effect of worm packing within the shell. Thus, the structure factor for the first population in model 2 (Eq. S5) is expressed as:

$$S_1(q) = \frac{1}{1 + 2A_2 \int_0^{\infty} F_1(q, r_{11})^2 \Psi_1(r_{11}) dr_{11}} \qquad \text{S6}$$

where A_2 is an effective virial coefficient.

The experimental SAXS pattern obtained for the 1.0 % w/w diblock copolymer worms prepared in *n*-dodecane can be satisfactorily fitted using the worm model (model 1), see Eq. S3 and Figure 4a. The resulting structural parameters (Table S3) are consistent with SAXS data recently reported for a worm dispersion with an identical target copolymer composition.[1] In particular, the worm contour length obtained in the present work (L_w = 591 ± 9 nm) is close to the lower limit estimated earlier (L_w ~ 600 nm), based on SAXS patterns that were truncated below q ~ 0.023 nm^{-1}. Moreover, the R_{sw}, b_w and L_w values are consistent with TEM observations (Figure 1 and Figure 2c). The copolymer *volume* fraction of 0.0069 obtained from SAXS analysis (Table S3) corresponds to a *mass* fraction of 0.01, which is in excellent agreement with the copolymer concentration of 1.0 % w/w used to prepare these emulsion droplets.

Model 2 (Eq. S5) produces a good fit to the SAXS data for the water droplets stabilised by the $PLMA_{16}$-$PBzMA_{37}$ worms in *n*-dodecane (Figure 4b). Unfortunately, there was no opportunity to collect SAXS data for this emulsion at lower *q* values (Figure 4b, $q < 0.08$ nm^{-1}). In order to obtain satisfactory data fits, several structural parameters, which are mainly associated with the high *q* region, were taken either from SAXS analysis of the worms *alone* (e.g., L_w and b_w) or from the volume-average droplet size distribution given by laser diffraction (e.g., the mean radius, R_{cs}, and associated standard deviation, σ_{21}, for the aqueous droplets, see supporting information) (Table S1).

Estimate of the specific surface area of a worm

Assume a worm of mean length L and mean width 2R, where R is the worm radius.

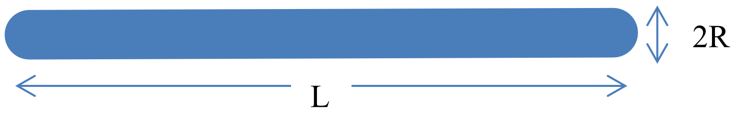

$$Specific\ surface\ area\ (As) = \frac{Area}{Mass}$$

$$As = \frac{2\pi RL + 4\pi R^2}{\rho.(\pi R^2 L + \frac{4}{3}\pi R^3)}$$

If L >> R, then the contribution from the spherical caps becomes negligible and hence

$$As = \frac{2\pi RL}{\rho.\pi R^2 L} \approx \frac{2}{\rho.R}$$

Thus this As value for worms is only ~ 33 % less than that for the corresponding spheres of mean radius R and identical copolymer density ρ, for which As = 3/ρ.R

Table S1. Variation in Pickering adsorption efficiency and mean droplet diameter calculated for various $PLMA_{16}$-$PBzMA_{37}$ worm copolymer concentrations.

**Initial copolymer worm concentration (wt %)**	**Final copolymer worm concentration (wt %)[a]**	**Adsorption efficiency (%)[a]**	**Number- average droplet diameter[b] (µm)**	**Volume- average droplet diameter[c] (µm)**
2.00	1.53	24	29 ± 12	50 ± 29
1.00	0.48	52	30 ± 10	49 ± 21
0.50	0.03	94	33 ± 16	55 ± 24
0.25	0.00	100	39 ± 13	67 ± 24
0.13	0.00	100	54 ± 25	100 ± 53
0.06	0.00	100	100 ± 40	117 ± 65

Determined using the following characterization techniques: (a) visible absorption spectroscopy, (b) optical microscopy and (c) laser diffraction. The standard deviation for the latter two techniques is also reported. This is calculated by taking the square root of the average of the squared differences of the values from their mean value.

Table S2. Volume-average droplet diameters determined by laser diffraction before and after heating to 95 °C for 90 minutes, along with the observed extent of demulsification.

Copolymer Morphology	Copolymer Concentration (wt %)	Fractional Surface Coverage, C	Initial Droplet Diameter (µm)	Final Droplet Diameter (µm)	Extent of demulsification (%)
Worms	0.50	1.33	55 ± 24	100 ± 51	17
	0.25	0.81	67 ± 29	123 ± 72	17
	0.13	0.62	100 ± 53	111 ± 54	17
	0.06	0.34	117 ± 65	demulsified	100
Spheres	0.50	0.61	199 ± 234	47± 18	17
	0.25	0.61	170 ± 223	171 ± 194	33
	0.13	0.61	139 ± 54	demulsified	100
	0.06	0.61	499 ± 336	demulsified	100

Table S3. Structural parameters obtained by SAXS analysis of a 1.0 % w/w $PLMA_{16}$-$PBzMA_{37}$ worm dispersion in *n*-dodecane (model 1) and a water-in-*n*-dodecane emulsion (water volume fraction = 0.50) stabilised using the same $PLMA_{16}$-$PBzMA_{37}$ worms (model 2).

Parameters	model 1	model 2
Population 1		
Worm contour length, L_w, nm	591 ± 9	591*
Kuhn length, b_w, nm	194 ± 6	194*
Worm core cross-section radius, R_{sw}, nm	5.9 ± 0.01	5.9 ± 0.1
R_{sw} standard deviation, σ_{11}, nm	0.74 ± 0.01	1.0 ± 0.08
Solvent volume fraction in the worm cores, x_{sol}	~0	~0
Radius of gyration of the corona block, R_g, nm	1.3 ± 0.1	1.1 ± 0.1
Copolymer volume fraction, c_1	0.0069 ± 0.00004	0.00056 ± 0.00004
Second virial coefficient (packing parameter), $A_2 \times 10^{16}$	-	1.68 ± 0.42
Population 2		
Core-shell radius, R_{cs}, nm	-	24500*
R_{cs} standard deviation, σ_{21}, nm	-	10500*
Shell thickness, T_{cs}, nm	-	12 ± 1.7
T_{cs} standard deviation, σ_{22}, nm	-	2.0**
Core-shell particles volume concentration, c_2	-	0.251 ± 0.005

N.B. Parameters denoted with an asterisk (*) were determined independently and were fixed during data fitting. The standard deviation for T_{cs} is directly related to that of R_{sw} ($\sigma_{22} = 2\sigma_{11}$).

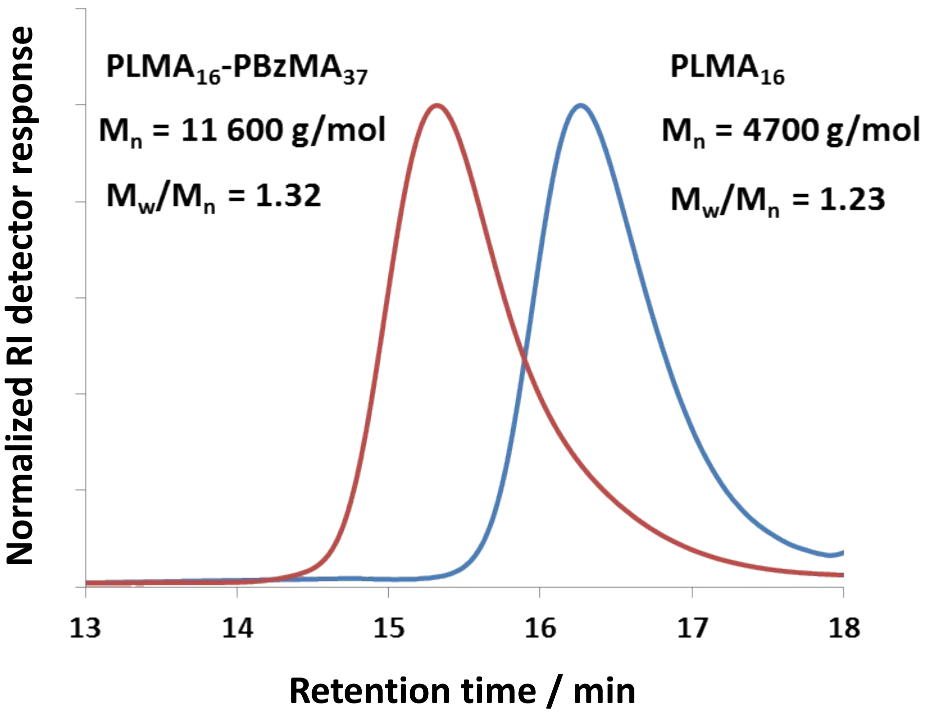

Figure S1. Gel permeation chromatography curves obtained for the precursor $PLMA_{16}$ macro-CTA and the resulting $PLMA_{16}$-$PBzMA_{32}$ diblock copolymer worms.

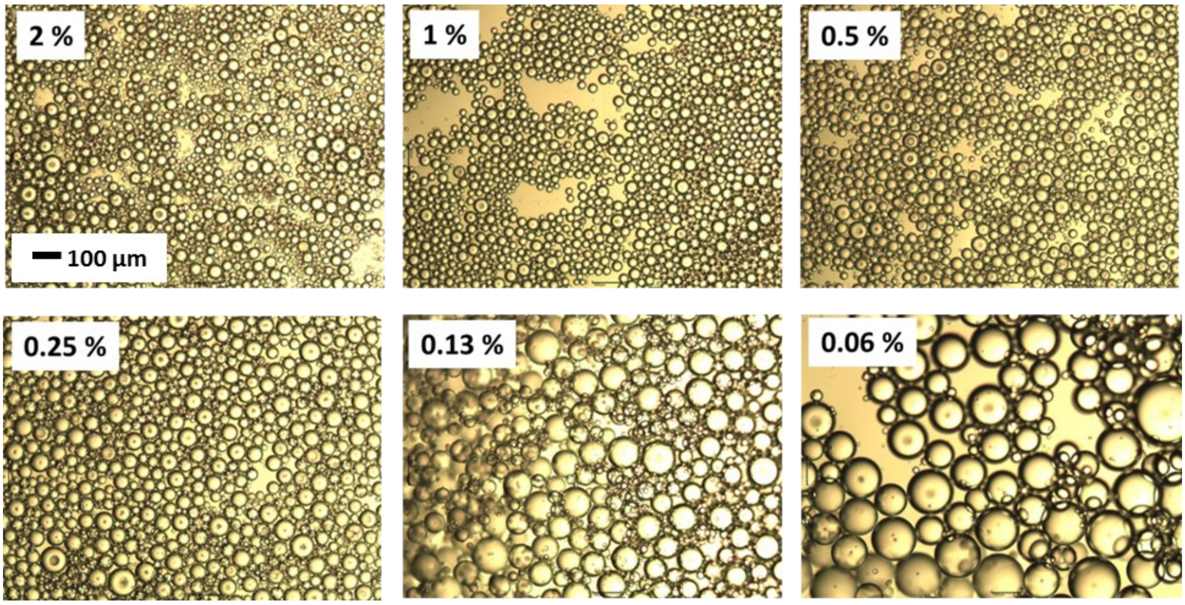

Figure S2. Representative optical microscopy images obtained for Pickering emulsions prepared using varying concentrations of $PLMA_{16}$-$PBzMA_{32}$ worms at 12 000 rpm for 2 minutes. The *n*-dodecane volume fraction was kept constant at 50 vol %.

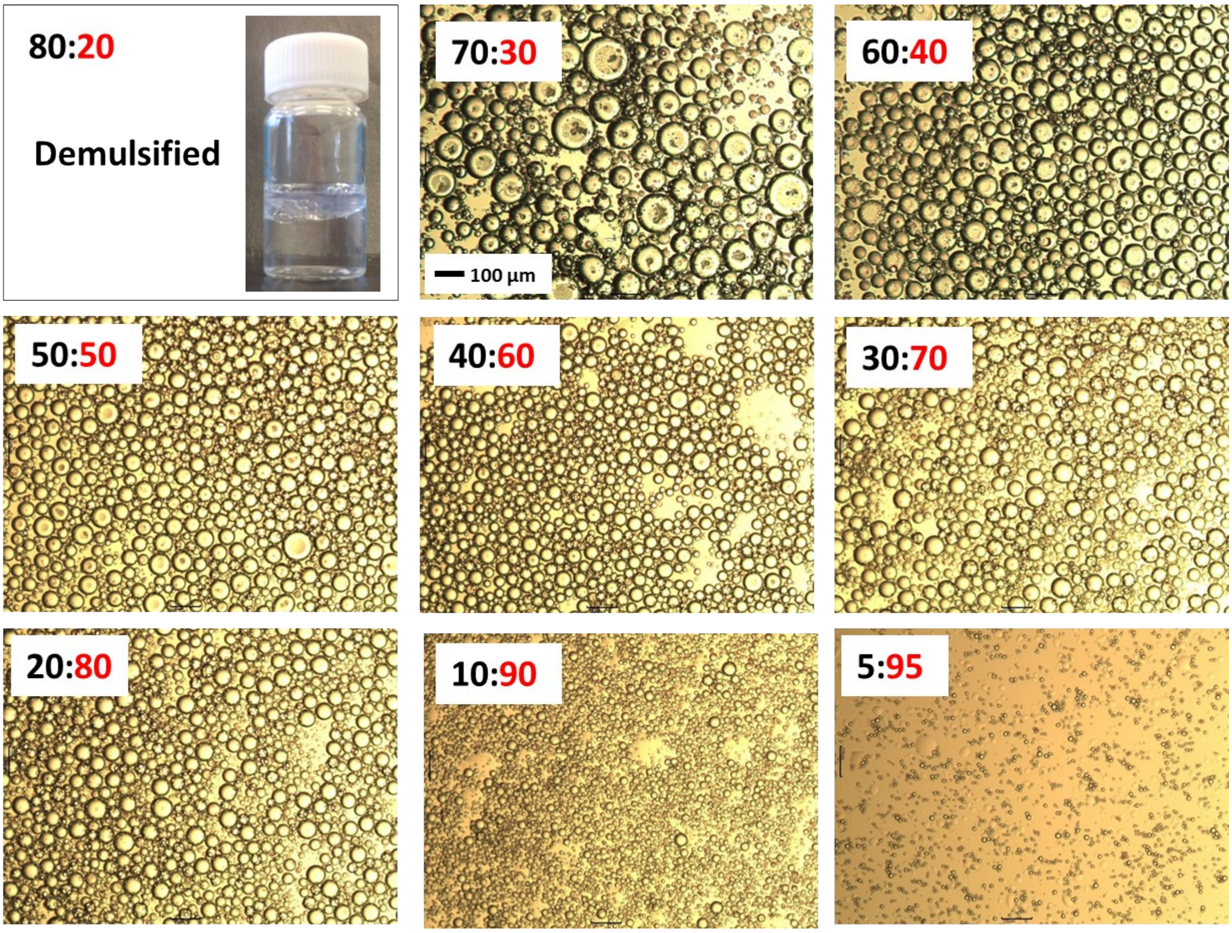

Figure S3. Optical microscopy images of stable w/o Pickering emulsions prepared using 0.50 % w/w L_{16}-B_{37} worms dispersed in *n*-dodecane, by systematically varying the w/o volume fraction from 80:20 to 5:95. A digital photograph of the demulsified sample obtained when using the 80:20 w/o ratio is also shown.

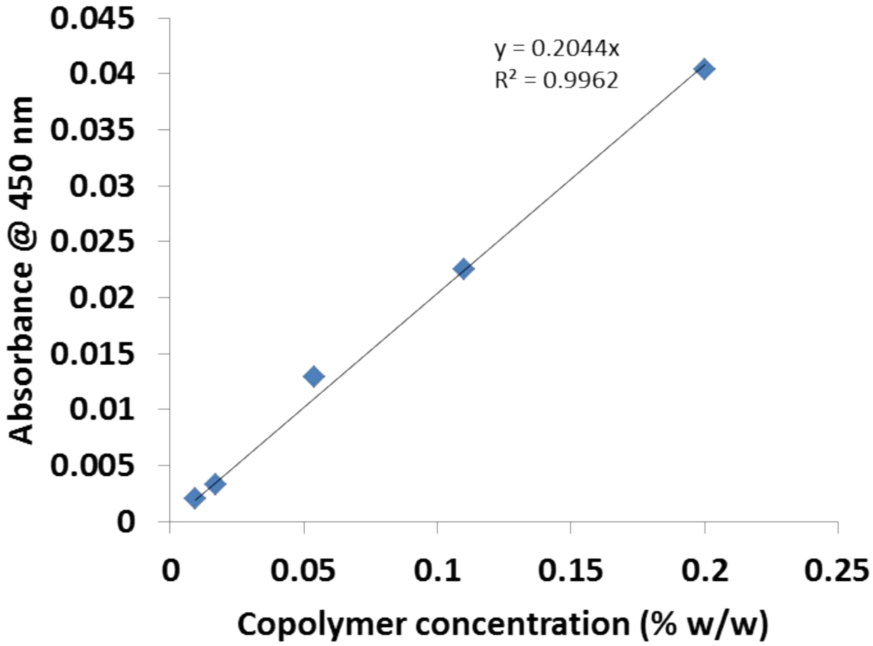

Figure S4. Linear Beer-Lambert plot constructed using visible absorption spectroscopy at an arbitrary wavelength of 450 nm. This calibration plot was used to estimate the supernatant concentration of non-adsorbed $PLMA_{16}$-$PBzMA_{37}$ worms remaining in the *n*-dodecane phase.

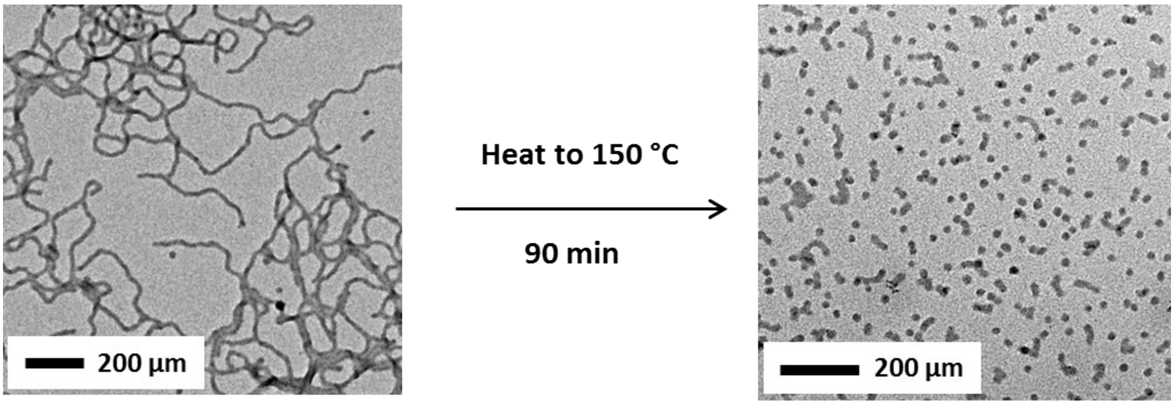

Figure S5. TEM images of ~ 0.1 % w/w $PLMA_{16}$-$PBzMA_{37}$ worms before (left) and after (right) heating to 150 °C for 90 minutes.

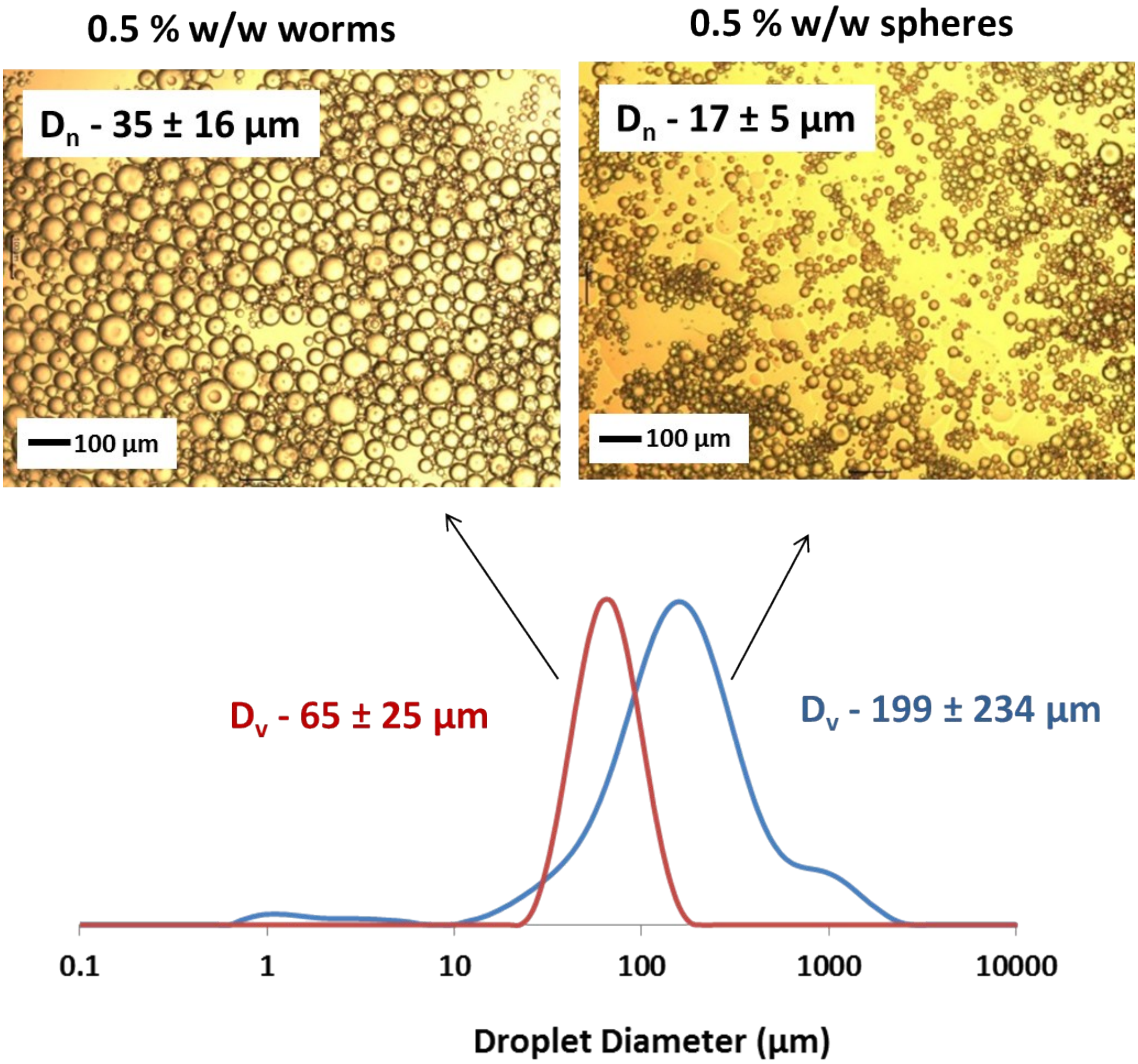

Figure S6. Optical microscopy and laser diffraction sizing data for Pickering emulsions formed using 0.50 % w/w $PLMA_{16}$-$PBzMA_{37}$ copolymer worms or spheres. Note the significant flocculation indicated by laser diffraction for the sphere-stabilised emulsion.

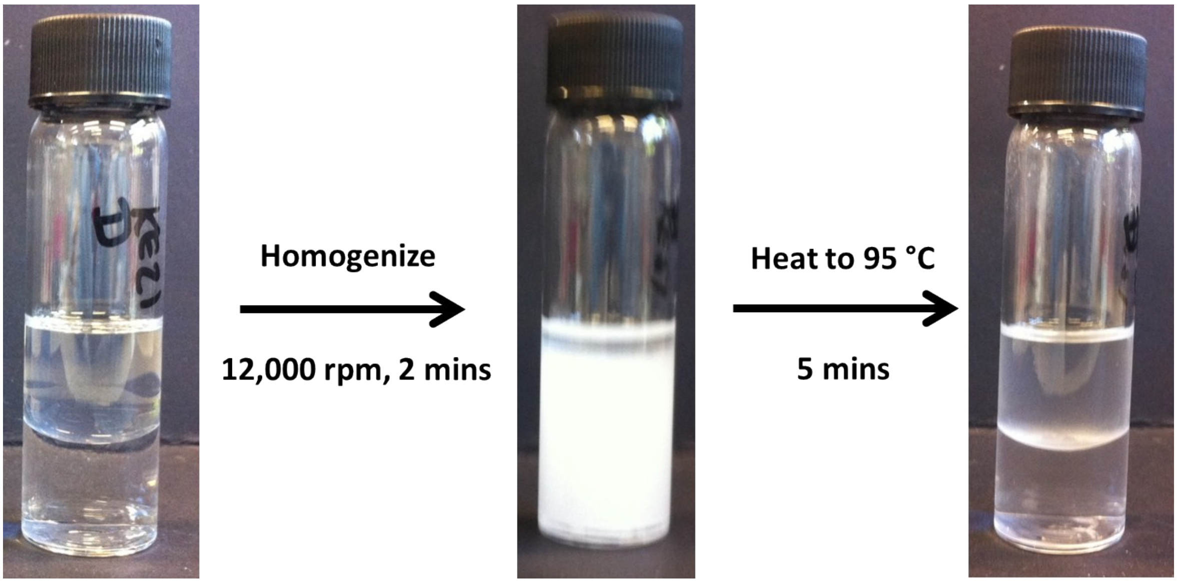

Figure S7. Digital photographs obtained for 0.06 % w/w $PLMA_{16}$-$PBzMA_{37}$ worms in *n*-dodecane plus an equal volume of water prior to homogenisation (left), the Pickering emulsion obtained after high shear homogenisation (center) and the phase separation caused by heating this emulsion up to 95°C for 5 min.

References.

1. L. A. Fielding, J. A. Lane, M. J. Derry, O. O. Mykhaylyk and S. P. Armes, *J. Am. Chem. Soc.*, 2014, **136**, 5790-5798.
2. J. S. Pedersen, *Journal of Applied Crystallography*, 2000, **33**, 637-640.
3. J. S. Pedersen and P. Schurtenberger, *Macromolecules*, 1996, **29**, 7602-7612.
4. J. A. Balmer, O. O. Mykhaylyk, A. Schmid, S. P. Armes, J. P. A. Fairclough and A. J. Ryan, *Langmuir*, 2011, **27**, 8075-8089.
5. J. S. Pedersen, *Advances in Colloid and Interface Science*, 1997, **70**, 171-210.
6. V. M. Garamus, J. S. Pedersen, H. Kawasaki and H. Maeda, *Langmuir*, 2000, **16**, 6431-6437.
7. R. Lund, V. Pipich, L. Willner, A. Radulescu, J. Colmenero and D. Richter, *Soft Matter*, 2011, **7**, 1491-1500.
